# *N*-Butyl-4,6-diphenyl­pyrimidin-2-amine

**DOI:** 10.1107/S160053681104147X

**Published:** 2011-10-12

**Authors:** Hoong-Kun Fun, Madhukar Hemamalini, Anita Hazra, Shyamaprosad Goswami

**Affiliations:** a X-ray Crystallography Unit, School of Physics, Universiti Sains Malaysia, 11800 USM, Penang, Malaysia; b Department of Chemistry, Bengal Engineering and Science University, Shibpur, Howrah 711 103, India

## Abstract

In the title compound, C_20_H_21_N_3_, the pyrimidine ring is inclined at dihedral angles of 51.57 (4) and 2.49 (4)° to the two phenyl rings. The dihedral angle between the two terminal phenyl rings is 50.44 (4)°. In the crystal, adjacent mol­ecules are linked *via* a pair of N—H⋯N hydrogen bonds, forming an inversion dimer with an *R*_2_^2^(8) ring motif. Furthermore, the crystal structure is stabilized by a weak π–π inter­action, with a centroid–centroid distance of 3.6065 (5) Å.

## Related literature

For biological applications of pyrimidine derivatives, see: Katrizky *et al.* (1982 ▶); Brown & Lyall (1964 ▶). For the synthesis, see: Goswami *et al.* (2009 ▶). For graph-set notation, see: Bernstein *et al.* (1995 ▶). For the stability of the temperature controller used in the data collection, see: Cosier & Glazer (1986 ▶).
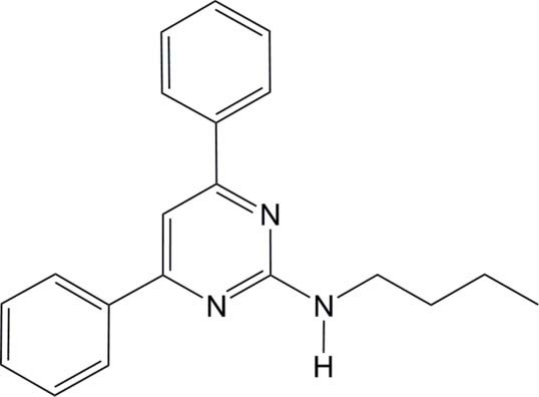


## Experimental

### 

#### Crystal data

C_20_H_21_N_3_
*M* *_r_* = 303.40
Triclinic, 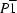

*a* = 8.1544 (1) Å
*b* = 9.5284 (1) Å
*c* = 11.3237 (2) Å
α = 77.090 (1)°
β = 74.152 (1)°
γ = 71.288 (1)°
*V* = 792.70 (2) Å^3^
*Z* = 2
Mo *K*α radiation
μ = 0.08 mm^−1^
*T* = 100 K
0.47 × 0.25 × 0.09 mm

#### Data collection

Bruker SMART APEXII CCD area-detector diffractometer
Absorption correction: multi-scan (*SADABS*; Bruker, 2009 ▶) *T*_min_ = 0.965, *T*_max_ = 0.993
26407 measured reflections
6933 independent reflections
5769 reflections with *I* > 2σ(*I*)
*R*_int_ = 0.031

#### Refinement

*R*[*F* ^2^ > 2σ(*F* ^2^)] = 0.048
*wR*(*F* ^2^) = 0.136
*S* = 1.04
6933 reflections
292 parameters
All H-atom parameters refined
Δρ_max_ = 0.56 e Å^−3^
Δρ_min_ = −0.31 e Å^−3^

### 

Data collection: *APEX2* (Bruker, 2009 ▶); cell refinement: *SAINT* (Bruker, 2009 ▶); data reduction: *SAINT*; program(s) used to solve structure: *SHELXTL* (Sheldrick, 2008 ▶); program(s) used to refine structure: *SHELXTL*; molecular graphics: *SHELXTL*; software used to prepare material for publication: *SHELXTL* and *PLATON* (Spek, 2009 ▶).

## Supplementary Material

Crystal structure: contains datablock(s) global, I. DOI: 10.1107/S160053681104147X/is2788sup1.cif

Structure factors: contains datablock(s) I. DOI: 10.1107/S160053681104147X/is2788Isup2.hkl

Supplementary material file. DOI: 10.1107/S160053681104147X/is2788Isup3.cml

Additional supplementary materials:  crystallographic information; 3D view; checkCIF report

## Figures and Tables

**Table 1:** Hydrogen-bond geometry (Å, °)

*D*—H⋯*A*	*D*—H	H⋯*A*	*D*⋯*A*	*D*—H⋯*A*
N3—H1*N*3⋯N1^i^	0.869 (15)	2.262 (15)	3.1249 (10)	172.4 (15)

Symmetry code: (i) 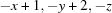.

**Fig. 1.**
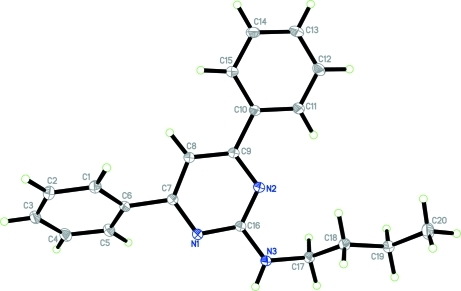
The molecular structure of the title compound, showing 50% probability displacement ellipsoids.

**Fig. 2.**
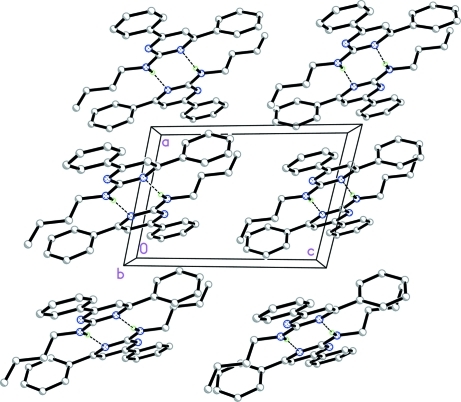
A crystal packing view of the title compound along the b axis.

## supplementary crystallographic information

### Comment

Substituted pyrimidine derivatives are utilized as antiviral and antifungal agents (Katrizky *et al.*, 1982; Brown & Lyall, 1964). 2-Butylamino-4,6-diphenyl pyrimidine has been synthesized by solid-phase microwave irradiation (Goswami *et al.*, 2009). The crystal structure of 2-butylamino-4,6-diphenylpyrimidine is reported here.

The molecular structure of the title compound is shown in Fig. 1. The pyrimidine (N1/N2/C7–C9/C16) ring is inclined at dihedral angles of 51.57 (4) and 2.49 (4)°, respectively, to the two phenyl (C1–C6 and C10–C15) rings. The corresponding angle between the two terminal phenyl (C1–C6 and C10–C15) rings is 50.44 (4)°.

In the crystal, (Fig. 2), the adjacent molecules are linked via a pair of N—H···N (Table 1) hydrogen bonds, forming an inversion dimer with an *R*_2_^2^(8) ring motif (Bernstein *et al.*, 1995).  The crystal structure is further stabilized by a weak π–π interaction between the pyrimidine (*Cg*1; N1/N2/C7–C9/C16) and phenyl (*Cg*3; C10–C15) rings [*Cg*1···*Cg*3^ii^ = 3.6065 (5) Å; (ii) 1 - *x*, 1 - *y*, -*z*].

### Experimental

A mixture of *S*-methylisothiourea sulphate (556 mg, 2 mmol), potassium carbonate (345 mg, 2.5 mmol) and butylamine (292 mg, 4 mmol) was irradiated at 450 Watt for 12 minutes in a microwave oven. The solid mass was washed with chloroform to remove the unreacted butylamine and then dried. The solid residue was then mixed with dibenzoylmethane (896 mg, 4 mmol) and again irradiated at 300 Watt for 6 minutes. Water was added to it and the contents were extracted with chloroform. The crude product was then purified through column chromatography (silica gel, 100–200 mesh) using 10% ethyl acetate in petroleum ether as an eluent to afford pure compound. The single crystal was grown by slow evaporation of a chloroform and methanol (3:1) solution (m.p. 65–66 °C).

### Refinement

All hydrogen atoms were located from a difference Fourier maps and refined freely [N—H = 0.869 (14) Å and C—H = 0.961 (15)–1.006 (12) Å]. The highest residual electron density peak is located at 0.68 Å from C3 and the deepest hole 1.26 Å located at from C16.

### Crystal data

**Table:**

C_20_H_21_N_3_	*Z* = 2
*M**_r_* = 303.40	*F*(000) = 324
Triclinic, *P*1	*D*_x_ = 1.271 Mg m^−^^3^
Hall symbol: -P 1	Mo *K*α radiation, λ = 0.71073 Å
*a* = 8.1544 (1) Å	Cell parameters from 8544 reflections
*b* = 9.5284 (1) Å	θ = 2.7–35.6°
*c* = 11.3237 (2) Å	µ = 0.08 mm^−^^1^
α = 77.090 (1)°	*T* = 100 K
β = 74.152 (1)°	Block, colourless
γ = 71.288 (1)°	0.47 × 0.25 × 0.09 mm
*V* = 792.70 (2) Å^3^	

### Data collection

**Table:**

Bruker SMART APEXII CCD area-detector diffractometer	6933 independent reflections
Radiation source: fine-focus sealed tube	5769 reflections with *I* > 2σ(*I*)
graphite	*R*_int_ = 0.031
φ and ω scans	θ_max_ = 35.0°, θ_min_ = 1.9°
Absorption correction: multi-scan (*SADABS*; Bruker, 2009)	*h* = −13→12
*T*_min_ = 0.965, *T*_max_ = 0.993	*k* = −15→15
26407 measured reflections	*l* = −18→17

### Refinement

**Table:**

Refinement on *F*^2^	Primary atom site location: structure-invariant direct methods
Least-squares matrix: full	Secondary atom site location: difference Fourier map
*R*[*F*^2^ > 2σ(*F*^2^)] = 0.048	Hydrogen site location: inferred from neighbouring sites
*wR*(*F*^2^) = 0.136	All H-atom parameters refined
*S* = 1.04	*w* = 1/[σ^2^(*F*_o_^2^) + (0.0772*P*)^2^ + 0.1237*P*] where *P* = (*F*_o_^2^ + 2*F*_c_^2^)/3
6933 reflections	(Δ/σ)_max_ = 0.001
292 parameters	Δρ_max_ = 0.56 e Å^−^^3^
0 restraints	Δρ_min_ = −0.31 e Å^−^^3^

### Special details

**Table:**

Experimental. The crystal was placed in the cold stream of an Oxford Cryosystems Cobra open-flow nitrogen cryostat (Cosier & Glazer, 1986) operating at 100.0 (1) K.
Geometry. All s.u.'s (except the s.u. in the dihedral angle between two l.s. planes) are estimated using the full covariance matrix. The cell s.u.'s are taken into account individually in the estimation of s.u.'s in distances, angles and torsion angles; correlations between s.u.'s in cell parameters are only used when they are defined by crystal symmetry. An approximate (isotropic) treatment of cell s.u.'s is used for estimating s.u.'s involving l.s. planes.
Refinement. Refinement of F^2^ against ALL reflections. The weighted R-factor wR and goodness of fit S are based on F^2^, conventional R-factors R are based on F, with F set to zero for negative F^2^. The threshold expression of F^2^ > 2σ(F^2^) is used only for calculating R-factors(gt) etc. and is not relevant to the choice of reflections for refinement. R-factors based on F^2^ are statistically about twice as large as those based on F, and R- factors based on ALL data will be even larger.

### Fractional atomic coordinates and isotropic or equivalent isotropic displacement parameters (Å^2^)

**Table:**

	*x*	*y*	*z*	*U*_iso_*/*U*_eq_	
N1	0.36098 (9)	0.87806 (7)	−0.01978 (6)	0.01301 (12)	
N2	0.37435 (8)	0.65917 (7)	0.13733 (6)	0.01297 (12)	
N3	0.48902 (9)	0.84810 (7)	0.14526 (6)	0.01494 (12)	
C1	0.25894 (11)	0.85602 (8)	−0.30090 (7)	0.01612 (14)	
C2	0.19425 (11)	0.94496 (9)	−0.40295 (8)	0.01783 (15)	
C3	0.08601 (11)	1.09047 (9)	−0.39389 (8)	0.01712 (14)	
C4	0.04519 (10)	1.14759 (8)	−0.28338 (8)	0.01701 (14)	
C5	0.11410 (10)	1.06024 (8)	−0.18232 (7)	0.01555 (14)	
C6	0.22075 (10)	0.91331 (8)	−0.19031 (7)	0.01279 (13)	
C7	0.28463 (10)	0.81869 (8)	−0.07941 (7)	0.01245 (12)	
C8	0.25519 (10)	0.67743 (8)	−0.03777 (7)	0.01350 (13)	
C9	0.30031 (9)	0.60099 (7)	0.07420 (7)	0.01186 (12)	
C10	0.26882 (9)	0.45275 (7)	0.13143 (7)	0.01231 (12)	
C11	0.31422 (10)	0.38330 (8)	0.24527 (7)	0.01509 (13)	
C12	0.28902 (11)	0.24334 (8)	0.29942 (8)	0.01732 (14)	
C13	0.21705 (11)	0.17083 (8)	0.24098 (8)	0.01694 (14)	
C14	0.17268 (11)	0.23799 (8)	0.12759 (8)	0.01749 (14)	
C15	0.19841 (10)	0.37773 (8)	0.07272 (8)	0.01567 (14)	
C16	0.40608 (10)	0.79250 (7)	0.08610 (7)	0.01233 (12)	
C17	0.51118 (10)	0.78847 (8)	0.27152 (7)	0.01481 (13)	
C18	0.66070 (11)	0.64437 (8)	0.28644 (7)	0.01577 (14)	
C19	0.68324 (11)	0.60211 (8)	0.42038 (8)	0.01700 (14)	
C20	0.82332 (13)	0.45432 (10)	0.44264 (10)	0.02491 (18)	
H1	0.3360 (16)	0.7506 (14)	−0.3075 (11)	0.021 (3)*	
H2	0.2272 (18)	0.9035 (15)	−0.4830 (12)	0.027 (3)*	
H3	0.0400 (17)	1.1531 (14)	−0.4640 (12)	0.022 (3)*	
H4	−0.0327 (17)	1.2473 (14)	−0.2754 (12)	0.024 (3)*	
H5	0.0862 (16)	1.1020 (13)	−0.1050 (11)	0.021 (3)*	
H8	0.2022 (17)	0.6372 (14)	−0.0856 (12)	0.025 (3)*	
H11	0.3622 (17)	0.4368 (14)	0.2855 (12)	0.025 (3)*	
H12	0.3224 (18)	0.1965 (14)	0.3804 (13)	0.027 (3)*	
H13	0.1961 (18)	0.0735 (15)	0.2800 (13)	0.029 (3)*	
H14	0.1241 (18)	0.1847 (15)	0.0865 (12)	0.028 (3)*	
H15	0.1669 (18)	0.4180 (14)	−0.0092 (12)	0.026 (3)*	
H17A	0.3985 (16)	0.7737 (13)	0.3224 (11)	0.017 (3)*	
H17B	0.5357 (16)	0.8685 (13)	0.3029 (11)	0.018 (3)*	
H18A	0.7692 (17)	0.6580 (14)	0.2288 (12)	0.023 (3)*	
H18B	0.6321 (17)	0.5604 (14)	0.2616 (12)	0.026 (3)*	
H19A	0.7185 (17)	0.6833 (14)	0.4430 (12)	0.023 (3)*	
H19B	0.5682 (17)	0.5940 (13)	0.4765 (12)	0.022 (3)*	
H20A	0.938 (2)	0.4555 (16)	0.3902 (14)	0.036 (4)*	
H20B	0.8407 (19)	0.4286 (16)	0.5301 (14)	0.034 (4)*	
H20C	0.7926 (19)	0.3696 (16)	0.4220 (13)	0.033 (3)*	
H1N3	0.5202 (19)	0.9295 (16)	0.1112 (13)	0.031 (3)*	

### Atomic displacement parameters (Å^2^)

**Table:**

	*U*^11^	*U*^22^	*U*^33^	*U*^12^	*U*^13^	*U*^23^
N1	0.0155 (3)	0.0116 (2)	0.0130 (3)	−0.00496 (19)	−0.0050 (2)	−0.00004 (19)
N2	0.0150 (3)	0.0112 (2)	0.0137 (3)	−0.00512 (19)	−0.0040 (2)	−0.00066 (19)
N3	0.0221 (3)	0.0124 (2)	0.0142 (3)	−0.0083 (2)	−0.0081 (2)	0.0009 (2)
C1	0.0197 (3)	0.0139 (3)	0.0158 (3)	−0.0044 (2)	−0.0064 (3)	−0.0018 (2)
C2	0.0220 (4)	0.0188 (3)	0.0143 (3)	−0.0066 (3)	−0.0067 (3)	−0.0010 (2)
C3	0.0175 (3)	0.0179 (3)	0.0167 (3)	−0.0071 (2)	−0.0070 (3)	0.0031 (2)
C4	0.0165 (3)	0.0142 (3)	0.0185 (4)	−0.0033 (2)	−0.0051 (3)	0.0013 (2)
C5	0.0167 (3)	0.0137 (3)	0.0150 (3)	−0.0033 (2)	−0.0035 (3)	−0.0008 (2)
C6	0.0142 (3)	0.0119 (3)	0.0131 (3)	−0.0053 (2)	−0.0042 (2)	0.0006 (2)
C7	0.0134 (3)	0.0118 (3)	0.0122 (3)	−0.0038 (2)	−0.0031 (2)	−0.0012 (2)
C8	0.0165 (3)	0.0120 (3)	0.0137 (3)	−0.0057 (2)	−0.0052 (2)	−0.0005 (2)
C9	0.0122 (3)	0.0108 (3)	0.0128 (3)	−0.0038 (2)	−0.0023 (2)	−0.0017 (2)
C10	0.0123 (3)	0.0109 (3)	0.0139 (3)	−0.0043 (2)	−0.0023 (2)	−0.0010 (2)
C11	0.0184 (3)	0.0142 (3)	0.0137 (3)	−0.0070 (2)	−0.0041 (3)	0.0002 (2)
C12	0.0210 (3)	0.0152 (3)	0.0152 (3)	−0.0077 (2)	−0.0032 (3)	0.0019 (2)
C13	0.0174 (3)	0.0122 (3)	0.0203 (4)	−0.0063 (2)	−0.0011 (3)	−0.0009 (2)
C14	0.0189 (3)	0.0144 (3)	0.0220 (4)	−0.0077 (2)	−0.0052 (3)	−0.0026 (3)
C15	0.0182 (3)	0.0134 (3)	0.0175 (3)	−0.0063 (2)	−0.0062 (3)	−0.0008 (2)
C16	0.0139 (3)	0.0109 (3)	0.0128 (3)	−0.0040 (2)	−0.0035 (2)	−0.0014 (2)
C17	0.0191 (3)	0.0131 (3)	0.0139 (3)	−0.0045 (2)	−0.0067 (3)	−0.0016 (2)
C18	0.0187 (3)	0.0137 (3)	0.0166 (3)	−0.0041 (2)	−0.0069 (3)	−0.0024 (2)
C19	0.0203 (3)	0.0147 (3)	0.0177 (4)	−0.0052 (2)	−0.0082 (3)	−0.0002 (2)
C20	0.0288 (4)	0.0190 (4)	0.0285 (5)	−0.0016 (3)	−0.0166 (4)	−0.0012 (3)

### Geometric parameters (Å, °)

**Table:**

N1—C7	1.3378 (9)	C10—C11	1.3996 (10)
N1—C16	1.3598 (9)	C10—C15	1.4024 (10)
N2—C9	1.3455 (9)	C11—C12	1.3923 (10)
N2—C16	1.3479 (9)	C11—H11	0.974 (13)
N3—C16	1.3502 (9)	C12—C13	1.3914 (11)
N3—C17	1.4532 (10)	C12—H12	0.995 (13)
N3—H1N3	0.869 (14)	C13—C14	1.3883 (11)
C1—C2	1.3930 (11)	C13—H13	0.980 (14)
C1—C6	1.3952 (11)	C14—C15	1.3933 (11)
C1—H1	1.006 (12)	C14—H14	0.983 (14)
C2—C3	1.3931 (11)	C15—H15	0.991 (13)
C2—H2	1.005 (13)	C17—C18	1.5281 (10)
C3—C4	1.3905 (12)	C17—H17A	0.976 (12)
C3—H3	0.967 (13)	C17—H17B	1.002 (12)
C4—C5	1.3942 (11)	C18—C19	1.5259 (11)
C4—H4	0.967 (13)	C18—H18A	0.973 (13)
C5—C6	1.3989 (10)	C18—H18B	1.015 (13)
C5—H5	0.983 (12)	C19—C20	1.5224 (11)
C6—C7	1.4870 (10)	C19—H19A	1.009 (13)
C7—C8	1.3977 (10)	C19—H19B	0.995 (13)
C8—C9	1.3942 (10)	C20—H20A	0.961 (15)
C8—H8	0.977 (13)	C20—H20B	1.003 (14)
C9—C10	1.4879 (10)	C20—H20C	1.007 (14)
			
C7—N1—C16	115.44 (6)	C13—C12—H12	120.7 (8)
C9—N2—C16	117.01 (6)	C11—C12—H12	119.1 (8)
C16—N3—C17	122.97 (6)	C14—C13—C12	119.61 (7)
C16—N3—H1N3	119.7 (9)	C14—C13—H13	120.0 (8)
C17—N3—H1N3	116.9 (9)	C12—C13—H13	120.4 (8)
C2—C1—C6	120.39 (7)	C13—C14—C15	120.42 (7)
C2—C1—H1	119.9 (7)	C13—C14—H14	118.8 (8)
C6—C1—H1	119.7 (7)	C15—C14—H14	120.8 (8)
C1—C2—C3	120.08 (7)	C14—C15—C10	120.50 (7)
C1—C2—H2	119.4 (7)	C14—C15—H15	116.3 (7)
C3—C2—H2	120.5 (7)	C10—C15—H15	123.2 (7)
C4—C3—C2	119.84 (7)	N2—C16—N3	117.51 (6)
C4—C3—H3	119.3 (7)	N2—C16—N1	126.16 (7)
C2—C3—H3	120.8 (7)	N3—C16—N1	116.33 (6)
C3—C4—C5	120.14 (7)	N3—C17—C18	115.74 (6)
C3—C4—H4	120.4 (8)	N3—C17—H17A	108.5 (7)
C5—C4—H4	119.4 (8)	C18—C17—H17A	110.1 (7)
C4—C5—C6	120.25 (7)	N3—C17—H17B	106.5 (7)
C4—C5—H5	119.7 (7)	C18—C17—H17B	108.5 (7)
C6—C5—H5	120.1 (7)	H17A—C17—H17B	107.3 (10)
C1—C6—C5	119.26 (7)	C19—C18—C17	110.79 (6)
C1—C6—C7	121.10 (6)	C19—C18—H18A	111.7 (7)
C5—C6—C7	119.58 (7)	C17—C18—H18A	108.8 (7)
N1—C7—C8	122.79 (7)	C19—C18—H18B	109.7 (7)
N1—C7—C6	117.19 (6)	C17—C18—H18B	109.1 (7)
C8—C7—C6	119.93 (6)	H18A—C18—H18B	106.6 (10)
C9—C8—C7	117.28 (6)	C20—C19—C18	113.18 (7)
C9—C8—H8	122.7 (8)	C20—C19—H19A	108.2 (7)
C7—C8—H8	120.0 (8)	C18—C19—H19A	109.3 (7)
N2—C9—C8	121.17 (6)	C20—C19—H19B	108.4 (7)
N2—C9—C10	116.16 (6)	C18—C19—H19B	109.2 (7)
C8—C9—C10	122.67 (6)	H19A—C19—H19B	108.4 (10)
C11—C10—C15	118.50 (6)	C19—C20—H20A	112.0 (9)
C11—C10—C9	119.78 (6)	C19—C20—H20B	112.9 (8)
C15—C10—C9	121.71 (7)	H20A—C20—H20B	106.1 (12)
C12—C11—C10	120.77 (7)	C19—C20—H20C	110.9 (8)
C12—C11—H11	121.4 (8)	H20A—C20—H20C	105.5 (12)
C10—C11—H11	117.8 (8)	H20B—C20—H20C	109.0 (11)
C13—C12—C11	120.19 (7)		
			
C6—C1—C2—C3	−2.07 (12)	C8—C9—C10—C11	−178.80 (7)
C1—C2—C3—C4	1.10 (12)	N2—C9—C10—C15	−177.66 (6)
C2—C3—C4—C5	0.79 (12)	C8—C9—C10—C15	2.87 (11)
C3—C4—C5—C6	−1.73 (12)	C15—C10—C11—C12	−0.40 (11)
C2—C1—C6—C5	1.13 (12)	C9—C10—C11—C12	−178.79 (7)
C2—C1—C6—C7	178.34 (7)	C10—C11—C12—C13	−0.37 (12)
C4—C5—C6—C1	0.76 (11)	C11—C12—C13—C14	0.84 (12)
C4—C5—C6—C7	−176.49 (7)	C12—C13—C14—C15	−0.53 (12)
C16—N1—C7—C8	−1.02 (11)	C13—C14—C15—C10	−0.24 (12)
C16—N1—C7—C6	175.47 (6)	C11—C10—C15—C14	0.71 (11)
C1—C6—C7—N1	132.94 (8)	C9—C10—C15—C14	179.06 (7)
C5—C6—C7—N1	−49.86 (10)	C9—N2—C16—N3	−176.52 (6)
C1—C6—C7—C8	−50.47 (10)	C9—N2—C16—N1	4.11 (11)
C5—C6—C7—C8	126.73 (8)	C17—N3—C16—N2	−13.28 (11)
N1—C7—C8—C9	3.34 (11)	C17—N3—C16—N1	166.16 (7)
C6—C7—C8—C9	−173.05 (6)	C7—N1—C16—N2	−2.90 (11)
C16—N2—C9—C8	−1.39 (10)	C7—N1—C16—N3	177.72 (6)
C16—N2—C9—C10	179.13 (6)	C16—N3—C17—C18	78.93 (9)
C7—C8—C9—N2	−2.05 (11)	N3—C17—C18—C19	174.06 (6)
C7—C8—C9—C10	177.40 (6)	C17—C18—C19—C20	176.63 (7)
N2—C9—C10—C11	0.68 (10)		

### Hydrogen-bond geometry (Å, °)

**Table:**

*D*—H···*A*	*D*—H	H···*A*	*D*···*A*	*D*—H···*A*
N3—H1N3···N1^i^	0.869 (15)	2.262 (15)	3.1249 (10)	172.4 (15)

Symmetry codes: (i) −*x*+1, −*y*+2, −*z*.
